# Mesenchymal stem cell-derived exosomes as a nanotherapeutic agent for amelioration of inflammation-induced astrocyte alterations in mice

**DOI:** 10.7150/thno.33872

**Published:** 2019-08-14

**Authors:** Panpan Xian, Yue Hei, Rui Wang, Tian Wang, Junle Yang, Jianying Li, Zhengli Di, Zhiqin Liu, Andrius Baskys, Weiping Liu, Shengxi Wu, Qianfa Long

**Affiliations:** 1 Mini-invasive Neurosurgery and Translational Medical Center, Xi'an Central Hospital, No. 161, West 5th Road, Xincheng District, Xi'an, 710003, P.R. China; 2 Department of Neurosurgery, Xijing Hospital, Fourth Military Medical University, No.17 Changle West Road, Xi'an, 710032, P.R. China; 3 Department of Neurobiology, School of Basic Medicine, Fourth Military Medical University, No.169 Changle West Road, Xi'an, 710032, P.R. China; 4 Department of Neurology, Xi'an Central Hospital, No. 161, West 5th Road, Xincheng District, Xi'an, 710003, P.R. China; 5 Graduate College of Biomedical Sciences, Western University of Health Sciences, Pomona, CA, 91766, USA; 6 Dr. Baskys is a Visiting Professor at the Mini-invasive Neurosurgery and Translational Medical Center, Xi'an Central Hospital.

**Keywords:** MSC-Exo, astrocyte alterations, calcium signaling, Nrf2-NF-κB signaling, Nanotherapy

## Abstract

Mesenchymal stem cell-derived exosomes (MSC-Exo) have robust anti-inflammatory effects in the treatment of neurological diseases such as epilepsy, stroke, or traumatic brain injury. While astrocytes are thought to be mediators of these effects, their precise role remains poorly understood. To address this issue, we investigated the putative therapeutic effects and mechanism of MSC-Exo on inflammation-induced alterations in astrocytes.

**Methods**: Lipopolysaccharide (LPS)-stimulated hippocampal astrocytes in primary culture were treated with MSC-Exo, which were also administered in pilocarpine-induced status epilepticus (SE) mice. Exosomal integration, reactive astrogliosis, inflammatory responses, calcium signaling, and mitochondrial membrane potentials (MMP) were monitored. To experimentally probe the molecular mechanism of MSC-Exo actions on the inflammation-induced astrocytic activation, we inhibited the nuclear factor erythroid-derived 2, like 2 (Nrf2, a key mediator in neuroinflammation and oxidative stress) by sgRNA (in vitro) or ML385 (Nrf2 inhibitor) in vivo.

**Results**: MSC-Exo were incorporated into hippocampal astrocytes as well as attenuated reactive astrogliosis and inflammatory responses in vitro and in vivo. Also, MSC-Exo ameliorated LPS-induced aberrant calcium signaling and mitochondrial dysfunction in culture, and SE-induced learning and memory impairments in mice. Furthermore, the putative therapeutic effects of MSC-Exo on inflammation-induced astrocytic activation (e.g., reduced reactive astrogliosis, NF-κB deactivation) were weakened by Nrf2 inhibition.

**Conclusions**: Our results show that MSC-Exo ameliorate inflammation-induced astrocyte alterations and that the Nrf2-NF-κB signaling pathway is involved in regulating astrocyte activation in mice. These data suggest the promising potential of MSC-Exo as a nanotherapeutic agent for the treatment of neurological diseases with hippocampal astrocyte alterations.

## Introduction

Mesenchymal stem cells (MSCs) are multipotent stromal cells, which can be obtained from several sources, including umbilical cord, bone marrow, and adipose tissue 1, 2. MSCs show minimal immunogenicity, have immunomodulatory properties and possess a multi-differentiation potential and culture expandability among their biological characteristics 3, 4. MSC-based therapies show promise in the treatment of inflammatory disorders including certain brain diseases. Available evidences suggest that therapeutic effectiveness is a consequence of the paracrine action of extracellular vesicles (EVs) and soluble factors secreted by MSCs 5, 6. Exosomes containing EVs of an endosomal origin (φ30-150 nm) have been shown to encapsulate messenger RNAs, microRNAs, proteins, and liposomes, these factors that are considered useful therapeutic agents, drug delivery tools, therapeutic targets, or disease biomarkers 6, 7. Furthermore, exosomes can be readily available for use as they are resistant to freezing and thawing, more importantly, they can cross the blood-brain barrier 8. Therefore, compared to intact cells, the use of exosomes for the treatment of neurological disorders may have advantages. Although several recent studies identified MSC-derived exosomes (MSC-Exo) as a robust anti-inflammatory agent for the treatment of brain injury 9, 10, their specific biological activity on target cells, such as pro-inflammatory stimuli-altered astrocytes, remains incompletely understood.

Astrocytes are characteristic star-shaped glial cells responsible for nutritional support, blood-brain barrier formation, extracellular ion balance, and synaptic remodeling 11, 12. Previous research showed that astrocytes are activated by inflammatory stimuli, damage-associated molecules, or reactive oxygen species (ROS) 13. Persistent astrocyte activation (termed astrogliosis and defined by upregulation of GFAP expression) can facilitate subsequent glutamate excitotoxicity, gap junction alteration, and mitochondrial dysfunction 12. Neuroinflammation and ischemia induce two different types of reactive astrocytes, referred to as A1 and A2 14. The A1 astrocytes (marked by an increase in C3 expression) loose most normal astrocyte functions, which is an important characteristic of the inflammation-induced alteration of astrocytes, and have been shown to be neurotoxic to neurons and destructive to synapses 14. Due to its antiproliferative and antiadhesive effects, CD81, a tetraspanin molecule implicated in membrane functions, antigen presentation, and cellular interactions, is a well-established regulator of astrogliosis caused by nerve injury 15, 16. Nuclear factor erythroid-derived 2, like 2 (Nrf2) is a basic leucine zipper transcription factor and is held in the cytoplasm by Kelch like-ECH-associated protein 1 (Keap1), which is involved in regulating astrocyte alterations in response to cell stress and inflammation 17, 18. There is an extensive interaction between Nrf2 and nuclear factor-κB (NF-κB, a mediator of inflammation) that has been shown to be involved in transcriptional crosstalk and modulation of antioxidant and anti-inflammatory pathways 19. Thus, regulation of the Nrf2-NF-κB signaling pathway could be an important strategy to ameliorate inflammation-induced astrocytic alterations.

It has been shown that astrocyte alterations are involved in the initiation and progression of neurological diseases, including temporal lobe epilepsy 20, Alzheimer's 21, and Parkinson's 22 diseases. The mechanisms that are implicated in the abnormal astrocyte-mediated pathological phenotypes include impairments in ion channel buffering 23, altered mitochondrial functions 24, reduced glutamate uptake 25, and impaired adenosine homeostasis 26. We have previously shown that by acting on astrocytes, MSC-Exo reduced inflammation, prevented abnormal neurogenesis, and attenuated memory impairment associated with status epilepticus (SE) 8, but the underlying mechanisms of these therapeutic effects were not clear. In this study, we investigated putative therapeutic effects of MSC-Exo on the reactive phenotype, inflammatory responses, aberrant calcium signaling, and mitochondrial dysfunction of lipopolysaccharide (LPS)-stimulated astrocytes or SE-induced hippocampal inflammation. We also inhibited Nrf2 by sgRNA (*in vitro*) or ML385 (Nrf2 inhibitor) *in vivo* to probe the molecular mechanism of MSC-Exo actions on the inflammation-induced astrocytic activation.

## Materials and Methods

### Animals

Animal experiments were conducted on 1-8-week-old C57B/6 male mice purchased from the Experimental Animal Center of Xi'an Jiaotong University. All animal procedures were performed in accordance with the guidelines for the Care and Use of Laboratory Animals of the National Institutes of Health 27. Animals were housed in a controlled environment with 12:12 h light / dark cycle with food and water provided *ad libitum*.

### MSCs preparation and culture conditions for obtaining exosomes

Human umbilical cord tissue was obtained from 5 healthy consenting mothers (age 25-30) using standard procedures performed according to the Guideline of the Ethical Committee of the Xi'an Central Hospital and in keeping with National Institutes of Health Guidelines. MSCs were obtained from enzymatic digestion of Wharton's Jelly tissue and expanded using complete culture medium (CCM) consisting of α-minimum essential medium (α-MEM; Gibco, New York, USA) and 16.5% fetal bovine serum (FBS) (Gibco, New York, USA). The 2nd or 3rd passage of MSCs were assessed for meeting the minimum criteria established by the Mesenchymal and Tissue Stem Cell Committee of International Society for Cellular Therapy, as described in our previous report 28. For the culture conditions preparing for exosome isolation, exosome-depleted fetal bovine serum (FBS) was prepared by 18h ultracentrifugation of regular FBS at 100,000g using an SW32Ti rotor (XPN-100, Beckman, California, USA) 29. The top layers of the FBS supernatant (approx. 9/10) were retained and used in the subsequent culture. When the 5th passage of MSCs reached about 50-70% confluency, the medium was replaced with CCM containing exosome depleted FBS. After 24-48 h, the supernatants were harvested and processed immediately to isolate exosomes or frozen at -80°C for storage.

### MSC-Exo isolation and characterization

Exosomes were isolated from the fresh or frozen cell culture supernatants as previously described 30. Briefly, cells were removed by centrifugations at 300g for 10 min (TX-400 rotor, ST16R, Thermo Fisher, Massachusetts, USA). Subsequently, supernatants were centrifuged at 2,000g for 10 min to remove smaller cellular debris and at 10,000g for 30 min to remove apoptotic bodies (F15-6x100y rotor, ST16R, Thermo Fisher, Massachusetts, USA). Exosomes were then harvested by ultracentrifugation at 100,000g for 70 min in a swing rotor (SW32Ti, XPN-100, Beckman, California, USA). All centrifugation steps were performed at 4℃, and isolated exosomes were suspended in phosphate-buffered saline (PBS) and stored at -80℃. The protein content of exosome suspension was quantified using the bicinchoninic acid (BCA) assay (Beyotime, P0010, Beijing, China). The representative markers CD63 (1:1000, Abcam, California, USA) and CD81 (1:1000, Abcam, California, USA) of exosomes were assayed by Western blotting. Furthermore, the morphology and size distribution of exosomes were detected by transmission electron microscope (TEM) (JEM-1400, JEOL Ltd., Japan) and nanoparticle tracking analysis (Nanosight LM10, Malvern, Worchestershire, UK), respectively. The protein content of the MSC-Exo preparations was normalized to total protein content as quantified by BCA assay.

### Labeling of MSC-Exo

MSC-Exo were labeled with the C5 Maleimide-Alexa 594 (CM-A954; Invitrogen, A10256, California, USA) as previously described 31. CM-A954 (200 μg/ml - 2.5 µl) was added to 50μl MSC-Exo (1.5 mg/ml) for 60 min in the dark (25°C), exosome spin columns (MW3000, Invitrogen) were prepared according to manufacturer's instructions, and powdered resin was hydrated for 25 min at room temperature. Spin columns in the collection tubes were centrifuged (750g) for 2 min using AllegraX-15R (Beckman, USA), and the columns were placed in 1.5 ml tubes and centrifuged for 3 min (750g) to collect labeled MSC-Exo. Non-incorporated or excess dye was retained by the resin and controls involving CM-A954, but no MSC-Exo were performed in parallel to confirm dye retention by the column. To avoid dye-stained aggregates, the labeled MSC-Exo were filtered through a 0.22 μm syringe filter immediately before use.

### Isolation of primary astrocytes

Newborn mice were anesthetized with isoflurane, the bilateral hippocampus was dissected out in a sterile environment, and the tissues were digested using 0.25% trypsin for 10 min at 37°C. Subsequently, hippocampal fragments were suspended in Dulbecco's Modified Eagle Medium (DMEM) containing 10% FBS, 100 units/ml penicillin, 100 μg/ml streptomycin and 2 mM L-glutamine, and the fragments were made into single-cell suspensions by repeated pipetting. Finally, astrocytes were isolated using a cell strainer (REF352350, FALCON, New York, USA). Characterization of the astrocytes was done by immunofluorescence using glial fibrillary acidic protein (GFAP, 1:1000) (Millipore, Boston, MA, USA) as the biomarker. Iba1 (microglia marker, 1:1250) (Abcam, Cambridge, Massachusetts, USA) was used to examine the purity of astrocytes and rule out the presence of microglia in the collected cells. Each experiment was done on at least 5 samples and each determination was made in triplicate unless indicated otherwise.

### Establishment of the SE model in mice

Induction of SE was performed as described previously 8. Briefly, subcutaneous injection of scopolamine methyl nitrate (1 mg/kg, Sigma-Aldrich, S2250, CA, USA) was used to reduce the peripheral cholinergic effects of the pilocarpine. After 30 min, animals received an intraperitoneal injection of 290-320 mg/kg pilocarpine hydrochloride (Sigma-Aldrich, P6503, CA, USA) to induce SE. Seizures were terminated by a diazepam injection (10 mg/kg) 2 h after the onset of SE. The severity of convulsive responses was monitored by a video camera and classified according to the modified Racine scale 32. Mice that showed consistent stage 4-5 seizures were used for SE-induced hippocampal inflammation experiments and those that did not show consistent acute seizure activity (stage 0-3) or developed severe tonic-clonic seizures were excluded from the study (these animals were euthanized to avoid pain and distress). Animals that did not receive pilocarpine hydrochloride injections served as the naïve control.

### Cytotoxicity assay

Cell Counting Kit-8 (CCK-8) (Beyotime, Beijing, China) was used to measure cell viability. Astrocytes were cultured at a density of 5×10^3^ cells per well in 96-well plates, divided into five groups and treated with PBS, 0.5 μg/ml, 1 μg/ml, 1.5 μg/ml and 2 μg/ml LPS for 3 h (n=9 samples in each group). Astrocytes were also divided into another set of six groups treated with PBS, LPS, 5 μg/ml, 10 μg/ml, 30 μg/ml and 50 μg/ml MSC-Exo for 12 h (n=9 samples in each group). These cells were treated with 1 μg/ml LPS for 3 h before switching to DMEM as in the previous report 33. The culture medium was removed and 10 μl CCK-8 solution mixed with 100 μl DMEM was added into each well for incubation for 2 h. Cell viability was estimated by measuring optical density (OD) of the astrocytes using a microplate reader (Bio-Rad Laboratories Inc., Hercules, California, USA) at 450 nm.

### *Nrf2* inhibition

For knockdown of* Nrf2 in vitro*, serum-free DMEM was added to hippocampal astrocytes that reached approximately 50% confluence in 6-well plates. The astrocytes were infected with U6-sgRNA-EF1a-Cas9-FLAG-P2A-EGFP lentiviral particles (Genechem Co., Ltd., Shanghai, China) for 12 h (37℃, 5% CO_2_) followed by DMEM + 10% FBS culture for 3-4 d. Puromycin dihydrochloride (Sigma-Aldrich, P9620, CA, USA) was used to select the stable clones expressing the *Nrf2* sgRNA (sgRNA sequence: AGAATTCCTCCCAATTCAGC), and the *Nrf2* knockdown astrocytes were expanded for subsequent experiments. Hippocampal astrocytes were also transduced with CON251 lentiviral particles (Genechem Co., Ltd., Shanghai, China) (sgRNA sequence: CGCTTCCGCGGCCCGTTCAA) as the negative control (NC, n=5 samples). The transfection efficiency was visualized via fluorescence microscopy (Leica, DMi8, Germany) and quantitatively assessed by Western blotting. *Nrf2* knockdown astrocytes received LPS stimulation only in the sgRNA+LPS group (n=5 samples), and both LPS and MSC-Exo treatment in the sgRNA+LPS+MSC-Exo group (n=5 samples).

To determine the regulatory role of *Nrf2 in vivo*, ML385 (*Nrf2* inhibitor; Medchem Express, Monmouth Junction, New Jersey, USA) was used to inhibit the *Nrf2* expression in mice as previously described with the following modifications 34, 35. Mice received a daily intraperitoneal injection of ML385 (30 mg/kg) dissolved in PBS with 5% Dimethyl Sulfoxide (DMSO) for 7 d. On day 3 of ML385 injection, SE was induced followed by treatment with PBS or MSC-Exo treatment ML385+SE+PBS (n=5) and ML385+SE+Exo (n=5) groups, respectively. Mice received an equivalent volume of the PBS with 5% DMSO instead of ML385 for the same duration as the vehicle control (n=5). Western blotting was employed to confirm the *Nrf2* inhibition.

### MSC-Exo administration

MSC-Exo were prepared at a concentration of 1.5 mg/ml in sterile PBS and stored at -80°C. For *in vitro* experiments, astrocytes were treated with 10 μg/ml exosomes (Figure S2A) for 12 h to ensure incorporation of the exosomes. The astrocytes were subsequently exposed to 1 μg/ml LPS for 3 h 33 as the LPS+MSC-Exo group. Astrocytes in Control, Control+MSC-Exo, and LPS groups were treated with PBS, 10 μg/ml exosomes, and 1 μg/ml LPS. For the *in vivo* experiments, mice that developed SE were randomly divided into the SE+PBS and SE+Exo groups and each mouse was injected with 20 µl PBS or MSC-Exo intraventricularly using a 25 μl Hamilton Syringe (Bregma: -0.6 mm, Midline: ±1.5 mm, Depth: 1.7 mm). The dose of injected MSC-Exo was determined by Western blotting (Figure S2B and C) as described in our previous study 8. The naïve control received an equivalent volume of the PBS and MSC-Exo as the Sham and Sham+Exo group, respectively.

### Tracking of administered MSC-Exo

MSC-Exo were tracked *in vitro* with 10 μg/ml CM-A954-labeled MSC-Exo that were added to the culture medium of the astrocytes and incubated for 12 h. Astrocyte culture slides (n=5) were then fixed with 4% paraformaldehyde (PH = 7.4) for immunostaining. For tracking of MSC-Exo *in vivo*, 20 μl CM-A954-labeled MSC-Exo were administered using methods described earlier 31. After the MSC-Exo injection, the mice (n=4) were transcardially perfused with paraformaldehyde saline solution (pH = 7.4) for 24 h. Cell or tissue processing, section cutting, storage of sections, and procedures employed for immunostaining were carried out as previously described 8, 28. Briefly, cell samples or tissue sections were labeled with primary antibodies for astrocyte marker GFAP (Millipore, MAB360, Massachusetts, USA) or s100-β (Abcam, ab218956, Massachusetts, USA). After the incubation with the secondary antibody A488 anti-mouse IgG (Thermo Fisher Scientific, A-21202, NY, USA) for 2.5 h, slides were mounted using 4', 6-diamidino-2-phenylindole (DAPI; Sigma-Aldrich, 32670, California, USA). Cultures or tissues processed without primary antibodies served as negative controls. Immunofluorescent images were detected using confocal microscopy (Olympus, FV10-ASW, Japan).

### Enzyme-linked immunosorbent assay (ELISA)

Astrocyte culture supernatants were collected from the Control, Control+MSC-Exo, LPS and LPS+MSC-Exo groups (n=5 each group) and measured using the Mouse Autoimmune Response ELISA Kits IL-1β (Cat# SEKM-0002), IL-6 (Cat# SEKM-0007), and TNFα (Cat# SEKM-0034) (Solarbio, Beijing, CHN). All ELISA procedures were performed according to the manufacturer's instructions, and the absorbance was measured with a microplate reader (Bio-Rad Laboratories Inc., Hercules, California, USA) at 450 nm. Each determination was made in triplicate.

### Calcium imaging

A calcium indicator Fluo-8 AM was used to detect the intracellular Ca^2+^ oscillations as previously described 23 with modifications. Cell samples including the Control, Control+MSC-Exo, LPS, and LPS+MSC-Exo groups (n=5 each group) were washed in serum- and phenol red-free DMEM containing 4 μM Fluo-8 AM (Abcam, ab142773, California, USA) plus 0.08% Pluronic F127 (Life Technologies, California, USA) for 20 min at 37°C, 5% CO_2_ to load the dye into the cells. Next, cultures were washed thrice and stored in artificial cerebrospinal fluid (ACSF) containing 124 mM NaCl, 25 mM NaHCO_3_, 2.5 mM KCl, 1 mM KH_2_PO_4_, 2 mM CaCl_2_, 2 mM MgSO_4_, and 10 mM glucose. Resting Ca^2+^ levels were recorded in ACSF for 30 s, and then 10 mM adenosine monophosphate (ATP) was used to stimulate the Ca^2+^ influx. The fluorescence of Fluo-8 AM was excited at the wavelength of 488 nm and measured every 1 second for 180 s using a confocal microscope (Olympus, FV3000, Japan). Calcium influx and resting Ca^2+^ levels were measured in individual astrocytes using the image analysis software Cellcens (Olympus, Japan). More than 90 cells for each experimental condition were analyzed using Igor Pro software (WaveMetrics, Oregon, USA) and results from at least three independent experiments were averaged. The intensity of excitation light and sampling frequency were kept as low as possible to minimize bleaching.

### JC-1 staining

The mitochondrial membrane potential (MMP) of astrocytes was measured using JC-1 (Solarbio, 3520-43-2, Beijing, CHN) staining, a dual-emission membrane potential-sensitive probe that exists as a green fluorescent monomer at a low MMP and forms aggregates with a fluorescent shift from green to red at a high MMP 36. Cell samples including the Control, Control+MSC-Exo, LPS, and LPS+MSC-Exo groups (n=6 each group) cultured in 24-well plates were washed thrice with PBS, and 5 mM JC-1 was added into the culture and incubated for 30 min at 37°C, 5% CO_2_. The change in fluorescence at 488 nm (green, excitation) and 594 nm (red, emission) was monitored by a confocal microscope (Olympus, FV3000, Japan), and the ratio of green to red fluorescence intensity was determined in this study. Each determination was an average of at least 3 independent experiments.

### Immunochemistry

Cell samples including the Control, Control+MSC-Exo, LPS, and LPS+MSC-Exo group (n=5 each group) were fixed with 4% paraformaldehyde (PH = 7.4) for immunostaining, and the animals in the Sham, Sham+Exo, SE+PBS, and SE+ Exo groups (n=5 per group) were sacrificed 4 d post-SE using isoflurane and processed for immunohistochemistry as previously reported 8, 28. Briefly, the primary antibodies consisted of mouse anti-GFAP (1:1000) (Millipore, MAB360, Massachusetts,USA), rabbit anti-Ki67 (1:500) (Thermo Fisher Scientific, PA5-19462, Illinois, USA), rabbit anti-nuclear factor-kappa B 65 (P-65, 1:500) (Thermo Fisher Scientific, MA5-15160, Illinois, USA), rabbit anti-C3 (1:100) (Thermo Fisher Scientific, PA5-21349, Illinois, USA), mouse anti-Nrf2 (1:100) (Abcam, Ab31163, California, USA), rabbit anti-CD81 (1:200) (Abcam, ab219209, California, USA), rabbit anti-IL-1β antibody (Abcam, ab9722, California, USA), or rabbit anti-TNFα (1:200) (Abcam, ab6671, California, USA) antibodies. After an overnight incubation with the respective primary antibody solution, cells or sections were washed thrice in PBS, and samples subjected to treatment with A488 anti-rabbit IgG (1:500) (Thermo Fisher Scientific, A-32790, New York, USA), or A594 anti-mouse IgG (1:500) (Thermo Fisher Scientific, A-32790, New York, USA) antibodies for 2 h at room temperature. DAPI was used to probe cell nuclei. Cell samples and hippocampal tissues were imaged using a confocal microscope (Olympus, FV10-ASW, Japan) or a fluorescent microscope (Leica, DMi8, Germany). Images were analyzed using the ImageJ Pro Plus V 6.0 (Bethesda, Maryland, USA).

### Western blotting

Cell samples were harvested from Control, Control+MSC-Exo, LPS, LPS+MSC-Exo, NC, sgRNA, sgRNA+LPS, or sgRNA+LPS+MSC-Exo groups (n=5 per group). Hippocampal tissues were collected at 24 h, 4 d, 7 d or 8 w post-SE from animals belonging to Sham, Sham+Exo, SE+PBS, SE+Exo, Vehicle, ML385, SE+ML385+PBS, or SE+ML385+Exo groups (n=5 per group per time point), and the samples or tissues were processed for Western blotting as previously described 28. Briefly, proteins were extracted using Radio-Immunoprecipitation Assay (RIPA) Lysis Buffer (Beyotime, P0013B, Beijing, China) and quantified using a BCA assay. Normalized protein samples were subjected to sodium dodecyl sulfate-polyacrylamide gel treatment, and transferred to nitrocellulose membranes (Millipore, MA, USA). Membranes were blocked using 5% skim milk in Tris Buffered saline Tween (TBST) at room temperature for 1.5 h and incubated with primary antibodies including CD81 (1:1000), GFAP (1:1000), C3 (1:1000), Nrf2 (1:1000), hemeoxygenase-1 (HO-1, 1:1000) (Proteintech, 10701-1-AP, Illinois, USA), kelch-like ECH-associated protein 1 (Keap1, 1:1000) (Proteintech, 10503-2-AP, Illinois, USA), nuclear factor-kappa B 65 (P-65, 1:1000) (Abcam, Ab16502, California, USA), phosphor NF-κBp-65 (p-P65, 1:2000) (Abcam, Ab86299, California, USA) or β-actin (1:100000) (Abclonal, AC026, Wuhan, China) overnight at 4°C, followed by washing with TBST for three times. Membranes were then incubated with the horseradish peroxidase-conjugated secondary antibodies (Thermo Fisher Scientific, New York, USA) at room temperature for 1.5 h. Labeled protein was detected using a Bio-Rad imaging system (Bio-Rad, Hercules, California, USA) and quantified using the Quantity One software package (West Berkeley, California, USA).

### Real-time quantitative PCR (qPCR)

Total RNA was isolated from the bilateral hippocampus of animals 4 d post-SE (Sham, Sham+Exo, SE+PBS, SE+Exo group, n = 5 per group) using TRIzol™ Reagent (Life Technologies, 15596026, New York, USA) according to the manufacturer's instructions, and the detailed procedures regarding qPCR in this study were performed as previously reported 28. RNA contents and the purity were measured by the ratio of absorbance at 260 / 280 nm. All the primer sequences including* GAPDH (Forward 5'-AAATGGTGAAGGTCGGTGTGAAC-3', Reverse 5'-CAACAATCTCCACTTTGCCACTG-3'), TNF-α (Forward 5'-ACTCCAGGCGGTGCCTATGT-3', Reverse 5'-GTGAGGGTCTGGGCCATAGAA-3'), IL-1β (Forward 5'-TCCAGGATGAGGACATGAGCAC-3', Reverse 5'-GAACGTCACACACCAGCAGGTTA-3'), and IL-6 (Forward 5'-CCACTTCACAAGTCGGAGGCTTA-3', Reverse 5'-TGCAAGTGCATCATCGTTGTTC-3')* were designed and optimized by TaKaRa (TaKaRa, Dalian, China). Reverse transcription was performed using the TaqMan® MicroRNA Reverse Transcription Kit (Thermo Fisher Scientific, 4366597, Massachusetts, USA) on a CFX Connect Real-Time PCR Detection System (Bio-Rad, California, USA). PCR products were visualized on a SYBR Safe stained agarose gel (Thermo Fisher Scientific, s33102, Massachusetts, USA) and the bands digitized using a Gel Doc™ EZ System (Bio-Rad, California, USA). Total RNA was used as a template in control PCR reaction, and normalization was based on the *GAPDH* gene expression. All data were obtained from at least three independent experiments and analyzed using Bio-Rad CFX 2.1 in triplicate.

### Behavioral testing

The cognitive function of animals in Sham, Sham+Exo, SE+PBS, and SE+ Exo group (n=8 per group) was assessed 8 w post-SE using the Morris Water Maze tracking system (Super Maze, XRXM101, China) as described in our previous report 37. The Morris Water Maze apparatus consisted of a circular pool (φ150cm) and a transparent platform. Briefly, each mouse was placed into water (23±1℃) from one starting position (North, South, West or East) chosen randomly, and the time to reach the platform (latency) that was located in the middle of the target quadrant was measured. Latency was recorded as 60 s if the mouse failed to find the platform within 60 s. The mice received training sessions from 52 to 55 d post-SE (4 trials per day), during which the escape latency to find the platform was recorded as the parameter for analyzing the spatial learning. After 24 h from the last training session, the platform was removed and the time spent in the target quadrant (% of total swim distance in target quadrant, PT) and platform crossings were recorded (4 trials) as the parameter for analyzing the spatial memory abilities and analyzed by Smart v2.5 software (Super Maze, China).

### Statistical analysis

Data were expressed as Mean ± SD. Multiple comparisons were analyzed using one-way analyses of variance (ANOVA) by Least Significant Difference (LSD) test, and repeated measures ANOVA was carried out to analyze the differences in learning curves and traveling distance among four groups after Bonferroni posttest using SPSS 19.0.0 and GraphPad Prism 7 software (Graphpad Prism, USA). *P* values of less than 0.05 were considered to be statistically significant.

## Results

### Characteristics of MSCs and exosomes

**Figure 1:**
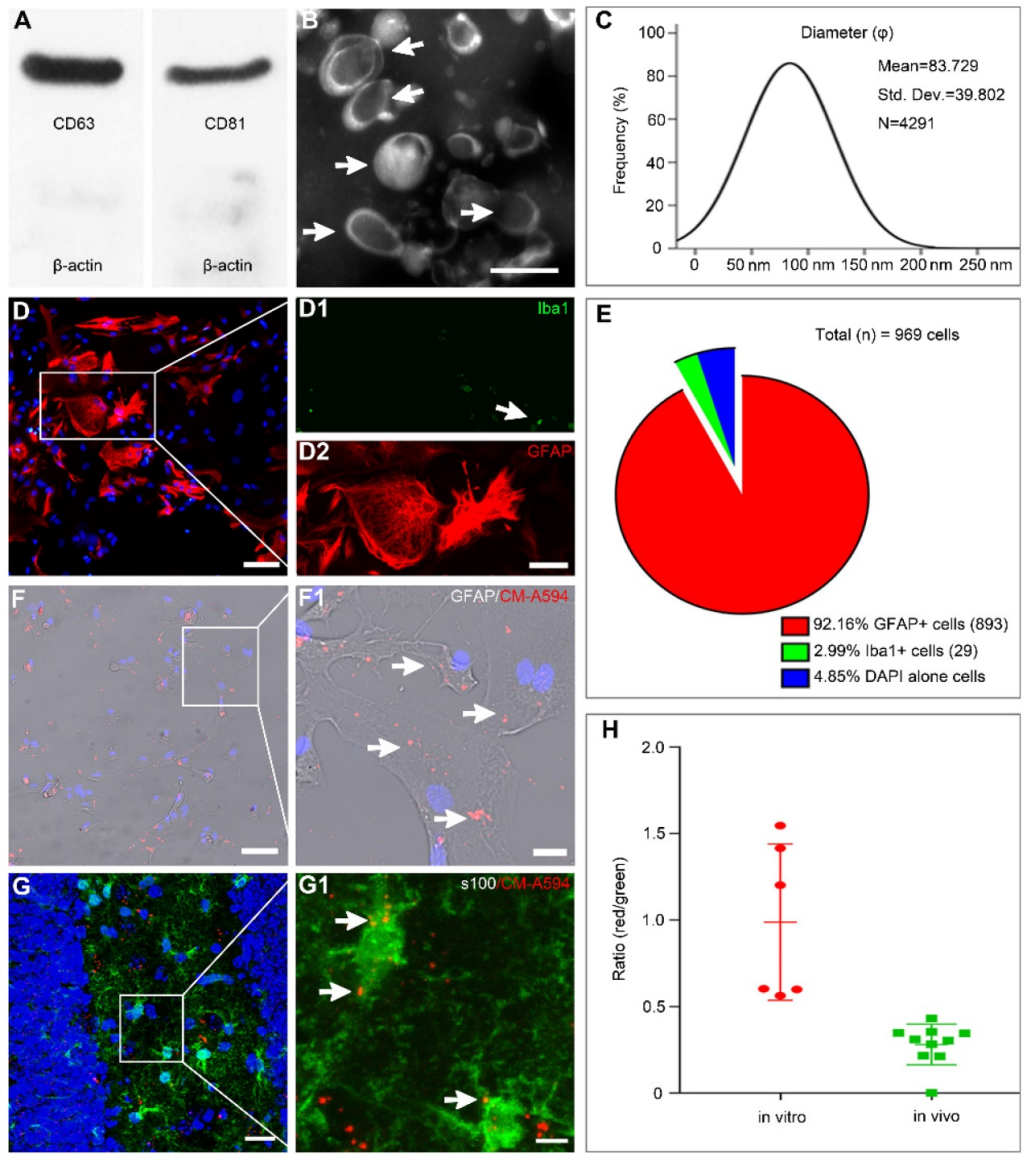
** MSC-Exo and astrocytes characterization, nanoparticles tracking.** (A) Western blots of protein expression in exosomes generated from the umbilical cord. (B) A representative TEM scanning image presents the morphology of MSC-Exo (arrowheads). (C) Size distribution of isolated MSC-Exo is shown in the graph on the right. (D) Cultured primary hippocampal astrocytes double-stained with the fluorescent antibody against Iba1 (D1, microglial marker, green) and GFAP (D2, astroglial marker, red). (E) Pie chart of cell samples composition. (F-G) Representative images showing the incorporation of MSC-Exo* in vitro* (F) and *in vivo* (G). Areas in squares are presented on the right at higher magnification (F1 and G1). MSC-Exo nanoparticles (red) in the soma and processes of GFAP+ (F1) or s100+ (G1)-stained astrocytes (arrowheads). (H) The histogram of the exosome uptake measured as the fluorescence intensity in primary cultured astrocytes (*in vitro*) and the hippocampus (*in vivo*). Scale bars: B, 200 nm; D = 50μm; D1 and D2, 25μm; F and G, 20μm; F1 and G1, 5μm.

Based on flow cytometry analysis, MSCs obtained from enzymatic digestion of Wharton's Jelly tissue were positive for CD73, CD90, and CD105 and negative for hematopoietic lineage markers such as CD34 and CD45. They showed a potential for differentiation into adipocyte, osteocyte, and chondrocyte lineages, as described in our recent report 28. We also found that MSC-Exo generated through a series of ultra-centrifugations were positive for classical exosomal markers such as CD63 and CD81 found on the surface of MSCs (Figure 1A). When the morphology and particle size of MSC-Exo were examined by TEM and nanoparticle tracking analysis, the results showed a classical “rim of a cup” morphology of MSC-Exo (Figure 1B), and a vast majority of exosomes derived from MSCs had an expected diameter that ranged from 30 to 150 nm (Figure 1C). These results indicated that the characteristics of MSC-Exo in our experiments met the typical criteria for exosomes.

### Characterization of astrocytes and MSC-Exo incorporation

Double immunostaining with GFAP/Iba1 was used to examine the composition of cultured astrocytes (Figure 1D). The majority of cells (92.16%) expressed GFAP with only a few cells showing Iba1+ immunostaining (2.99%, Figure 1E). For tracking the MSC-Exo *in vitro* and *in vivo*, the primary culture of astrocytes and SE model received CM-A594-labeled exosomes, and the cells or tissue samples were examined by GFAP (Figure 1F) or s100-β (Figure 1G) staining. Red MSC-Exo nanoparticles appeared to be in smaller clusters and were seen either within the cytoplasm or processes in the entire primary culture of astrocytes (Figure 1F1), as well as observed in hippocampal astrocytes of the mice (Figure 1G1). Statistical analysis showed that the fluorescence intensity of exosomal uptake was 0.9885 ± 0.1844 and 0.2812 ± 0.03721 in the cell samples and hippocampus, respectively. These results indicated that MSC-Exo can be incorporated into hippocampal astrocytes* in vitro* and *in vivo*.

### Treatment with MSC-Exo attenuated the LPS-induced cytotoxicity, reactive astrogliosis, and inflammatory responses *in vitro*

**Figure 2:**
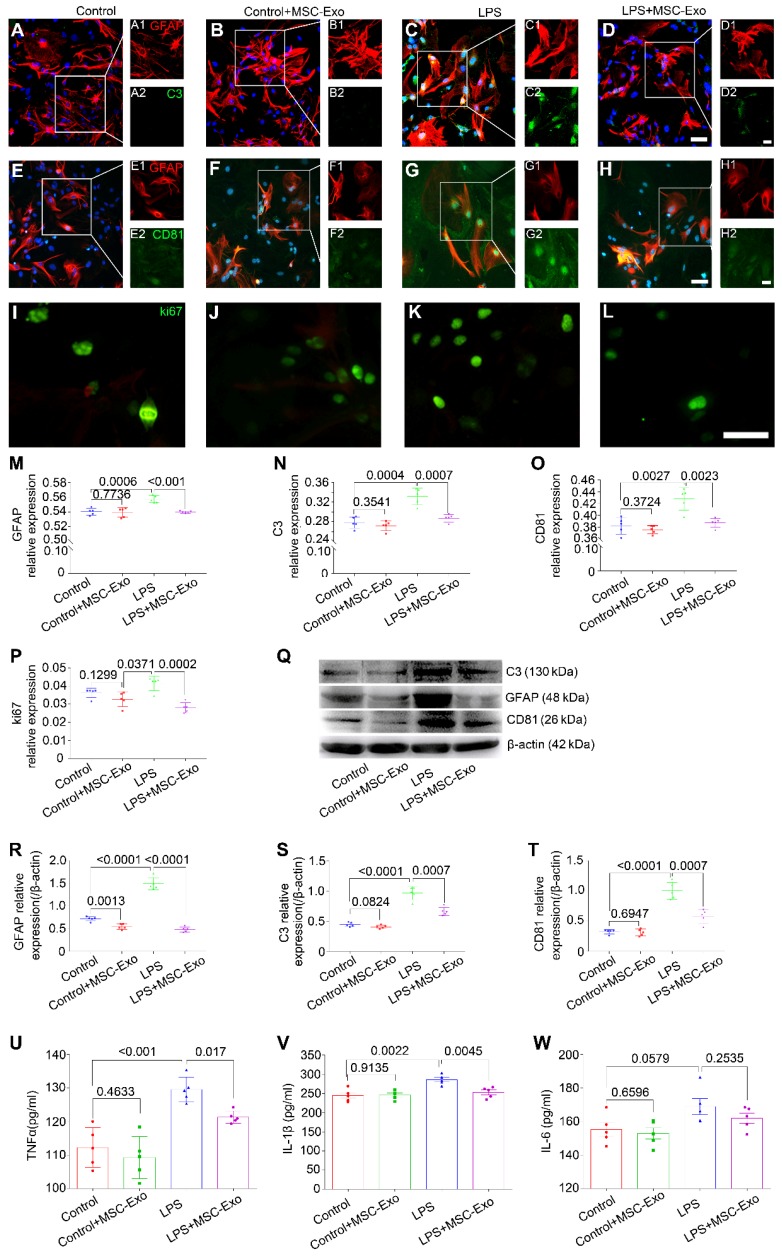
** MSCs-Exo protect against LPS-induced astrocytic activation and inflammatory responses* in vitro*.** Astrocytic activation and inflammatory responses were examined using immunohistochemistry, Western blotting, or ELISA. (A-L) Immunostaining images of GFAP (A-H, red, a marker of reactive astrocytes), C3 (A-D, green, a marker A1 astrocytes), CD81 (E-H, green, a regulator of astrogliosis), and ki67 (I-L, green, a marker of cell proliferation). (M-P) Statistical analysis of the fluorescence intensity of GFAP (M), C3 (N), CD81 (O), and ki67 (P) in the Control, Control+MSC-Exo, LPS, and LPS+MSC-Exo group. (Q) Western blots of the relative expression of GFAP (R), C3 (S), and CD81 (T) in each group. (U-W) Concentration of inflammatory cytokines secreted by astrocytes including TNF-α (U), IL-1β (V), and IL-6 (W) determined by ELISA. Histograms of statistical difference between groups are shown. Scale bars: A-H, 50 μm; I-L, A1-H1 and A2-H2, 20 μm.

The effect of MSC-Exo on LPS-induced alterations in astrocytes was determined by cytotoxicity, reactive astrogliosis, and inflammatory cytokine analysis. CCK-8 results showed that stimulation with different concentrations (0.5-2.0μg/ml) of LPS induced a remarkable decrease of cell viability in primary culture of astrocytes (Figure S2A) and MSC-Exo treatment significantly reduced this cytotoxicity (Figure S2B). Furthermore, immunofluorescence experiments (Figure 2A-L) showed that, compared to the LPS group, MSC-Exo treatment reduced the relative expression of GFAP (a reactive astrogliosis marker) (Figure 2M, *P* < 0.001), C3 (A1 astrocyte marker) (Figure 2N, *P* = 0.0007), CD81 (an essential regulator of astrocytic activation) (Figure 2O, *P* = 0.0023), and ki67 (cell proliferation marker) (Figure 2P, *P* = 0.0002). Western blotting (Figure 2Q) showed that there was a significant decrease in GFAP (Figure 2R, *P* < 0.0001), C3 (Figure 2S, *P* = 0.0007), and CD81 (Figure 2T, *P* = 0.0007) protein expression in the LPS+MSC-Exo group compared to the LPS group. We next used ELISA to examine the anti-inflammatory response of MSC-Exo on LPS-induced astrocytes. Data showed that compared to the LPS group, MSC-Exo treatment remarkably attenuated the secretion of TNFα (Figure 2U, *P* = 0.017) and IL-1β (Figure 2V, *P* = 0.0045), but not IL-6 in the culture medium (Figure 2W, *P* = 0.2535). These results suggested that LPS-induced cytotoxicity, reactive astrogliosis, and inflammatory responses can be significantly reduced by MSC-Exo.

### MSC-Exo treatment ameliorates LPS-induced aberrant calcium signaling and mitochondrial dysfunction in primary culture of astrocytes

**Figure 3:**
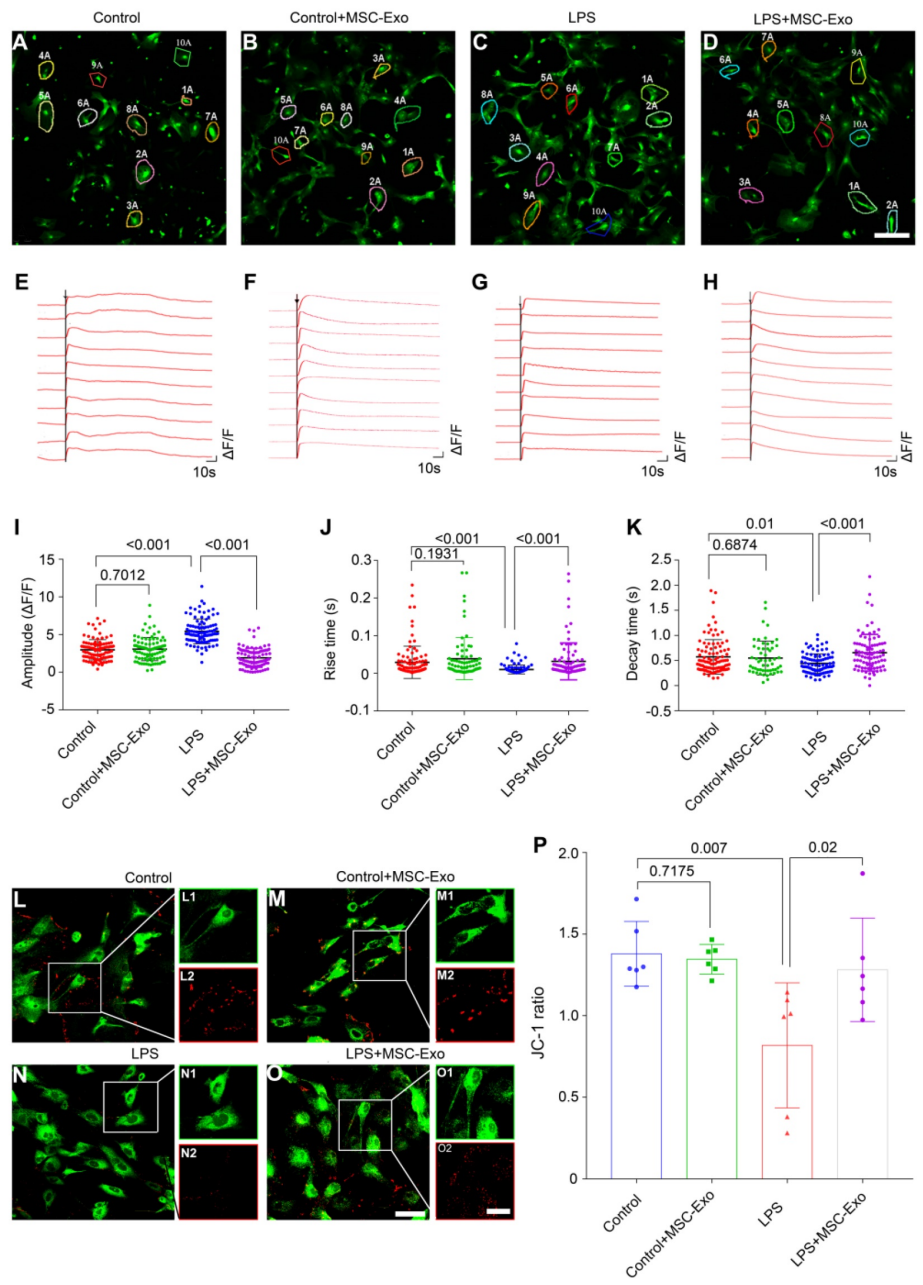
** MSC-Exo treatment ameliorates LPS-induced aberrant calcium signaling and mitochondrial dysfunction in the primary culture of hippocampal astrocytes.** A calcium indicator Fluo-8 AM was used to detect the intracellular Ca^2+^ oscillations in cultured primary astrocytes. (A-D) Examples of Ca^2+^ imaging in cultured primary astrocytes obtained with confocal microscopy using Fluo-8 AM in Control (A), Control+MSC-Exo (B), LPS+PBS (C), and LPS+MSC-Exo groups (D). (E-H) Igor software assay presents the first phase (30 s) and second phase (150 s) of the intracellular Ca^2+^ oscillations within 10 cells selected at random from each group. (J-K) Statistical analysis of the amplitude (ΔF/F) (I), rise time (J), and decay time (K) of the intracellular Ca^2+^ oscillations after adding ATP in each group. (L-O) Representative images of the mitochondrial membrane potential in the primary culture of hippocampal astrocytes using JC-1 staining. Green fluorescence of the monomeric form of JC-1 in the cytosol after mitochondrial membrane depolarization (L1-O1), red fluorescence of the potential-dependent aggregation in the mitochondria (L2-O2) in each group. (P) The histogram of the ratio of JC-1 fluorescence (Red / Green) in the primary culture of hippocampal astrocytes. Statistical difference between groups is shown in all histograms. Scale bars: A-D, 50 μm; L-O, L1-O1 and L2-O2, 100 μm.

We used calcium imaging to investigate calcium signaling alteration in the primary culture of astrocytes. Our data showed that astrocytes had different fluorescence properties in various groups (Figure 3A-D). Following the ATP stimulation, there was a first phase Ca^2+^ response consisting of a sharp peak representing a transient, large increase in intracellular Ca^2+^, followed by a second phase response of a slowly declining intracellular Ca^2+^ concentration in the Control (Figure 3E), Control+MSC-Exo (Figure 3F), LPS (Figure 3G), and LPS+MSC-Exo (Figure 3H) groups. Statistical analysis revealed that, compared to the Control group, the LPS group had an increased amplitude (ΔF/F) of intracellular Ca^2+^ oscillations after adding ATP, while no such changes were detected in the Control+MSC-Exo group (Figure 3I). MSC-Exo treatment (LPS+MSC-Exo) significantly reduced the Ca^2+^ influx compared to the LPS group (Figure 3I, *P* < 0.001). Also, LPS stimulation (LPS group) resulted in a faster change of the response rise time (Figure 3J, *P* < 0.001) as well as the decay time (Figure 3K, *P* = 0.01) compared to the Control group. These changes were not observed in the Control+MSC-Exo group (Figure 3J, *P* = 0.1931; Figure 3K, *P* = 0.6874). Remarkably, MSC-Exo treatment reversed the rate of Ca^2+^ oscillations that were observed in the LPS group (Figure 3J and K). As mitochondrial permeability is critical for the inflammation-induced astrocytic activation, the MMP of astrocytes in primary culture was further examined using the mitochondrion-specific lipophilic cationic fluorescence dye JC-1 (Figure 3L-O). Statistical analysis revealed a significant reduction of JC-1 ratio in the LPS group (Figure 3P, *P* = 0.007), which was reversed by the administration of MSC-Exo (Figure 3P, *P* = 0.02). No significant differences were found between the Control and Control+MSC-Exo groups (Figure 3P, *P* = 0.7175). Taken together, these data showed that MSC-Exo treatment ameliorates LPS-induced aberrant calcium signaling and mitochondrial dysfunction in the primary culture of hippocampal astrocytes.

### sgRNA knockdown of *Nrf2* decreases the inhibition of astrocytic activation by MSC-Exo *in vitro*

Western blotting and immunofluorescence were used to explore whether the Nrf2-NF-κB signaling pathway participated in the inhibition of astrocytic activation.

**Figure 4:**
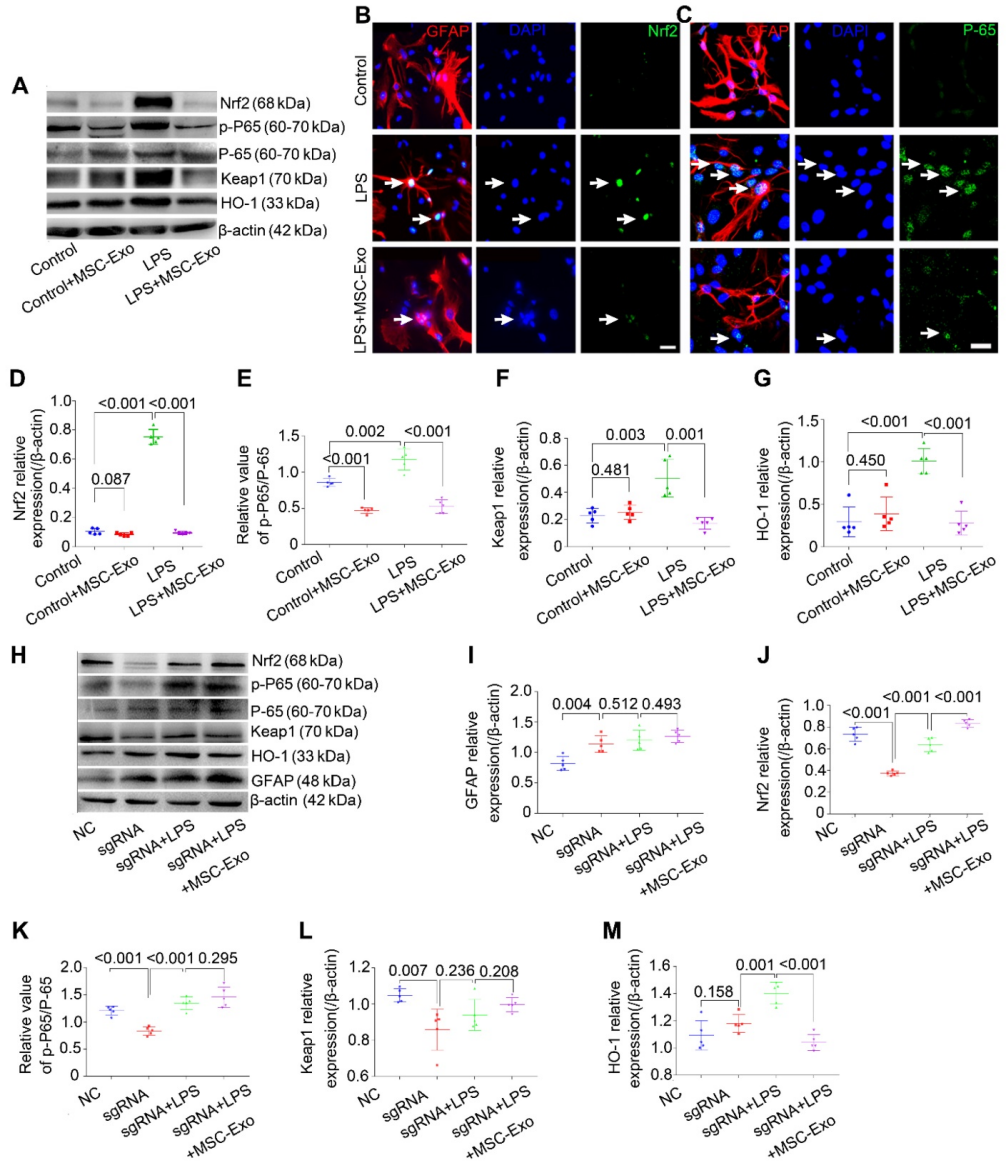
** sgRNA knockdown of Nfr2 decreases the inhibition of astrocytic activation by MSC-Exo *in vitro*.** Nrf2-NF-κB signaling in primary culture of astrocytes. (A) Western blotting of protein expression of Nrf2-NF-κB signaling in primary cultured astrocytes. (B-C) Representative images of the nuclear translocation of the Nrf2 (B, white arrow) and P-65 (C, white arrow) in LPS-induced astrocytes. (D-G) Statistical analysis of the relative expression of Nrf2 (D), Keap1 (F), HO-1 (G), and the value of p-P65/P-65 (E, represents NF-κB signaling activation) in each group. (H) Western blots of the relative expression of GFAP (I), Nrf2 (J), p-P65/P-65 (K), Keap1 (L), and HO-1 (M) in NC (Negative control), sgRNA, sgRNA+LPS, and sgRNA+LPS+MSC-Exo groups. Statistical difference between groups is shown in all histograms. Scale bars: B and C, 30μm.

Western blotting (Figure 4A) showed that LPS significantly increased the expression of antioxidant (Nrf2, Keap1, HO-1) and inflammatory (p-P65/P-65, NF-κB activation and inflammation marker) proteins (Figure 4D-G, *P* < 0.01, vs. Control group), whereas MSC-Exo treatment reversed these changes (Figure 4D-G, *P* < 0.01). Besides, NF-κB activation was reduced in the Control+MSC-Exo group (Figure 4E, *P* < 0.0001, vs. Control group). We used immunofluorescence to further examine the nuclear translocation of antioxidant and inflammatory factors. Our data showed that MSC-Exo treatment inhibited the nuclear translocation of Nrf2 and P-65 (Figure 4B, C) in response to LPS stimulation in cultured astrocytes.

To determine the inhibitory mechanism of the LPS-induced astrocytic activation by MSC-Exo inhibition, *Nrf2* gene in the primary cultured astrocytes was knocked down with sgRNA. Western blotting (Figure 4H) showed that knockdown of *Nrf2* (Figure 4J, *P* < 0.001) decreased the expression of NF-κB (Figure 4K, *P* < 0.001), Keap1 (Figure 4L, *P* < 0.0077), and HO-1 (Figure 4M, *P* = 0.0439) in the astrocytes compared to the NC group. While LPS stimulation upregulated Nrf2, NF-κB, and HO-1 expression compared with the sgRNA group (Figure 4J, K, *P* < 0.01), it did not change Keap1 expression (Figure 4L, *P* = 0.2367, vs. sgRNA group). Notably, the markers of oxidation (Nrf2, Keap1 and HO-1) and inflammation (p-P65/P-65 and GFAP) were not affected by MSC-Exo administration in the LPS-induced Nrf2 knockdown astrocytes (Figure 4I-M,* P* > 0.05, vs. sgRNA+LPS group). Taken together, these data suggested that LPS-induced astrocytic activation is prevented by MSC-Exo treatment and that the prevention is dependent on NF-κB-Nrf2 signaling pathway.

### Intraventricular injection of MSC-Exo reduces the hippocampal reactive astrogliosis in a mouse model of SE

**Figure 5:**
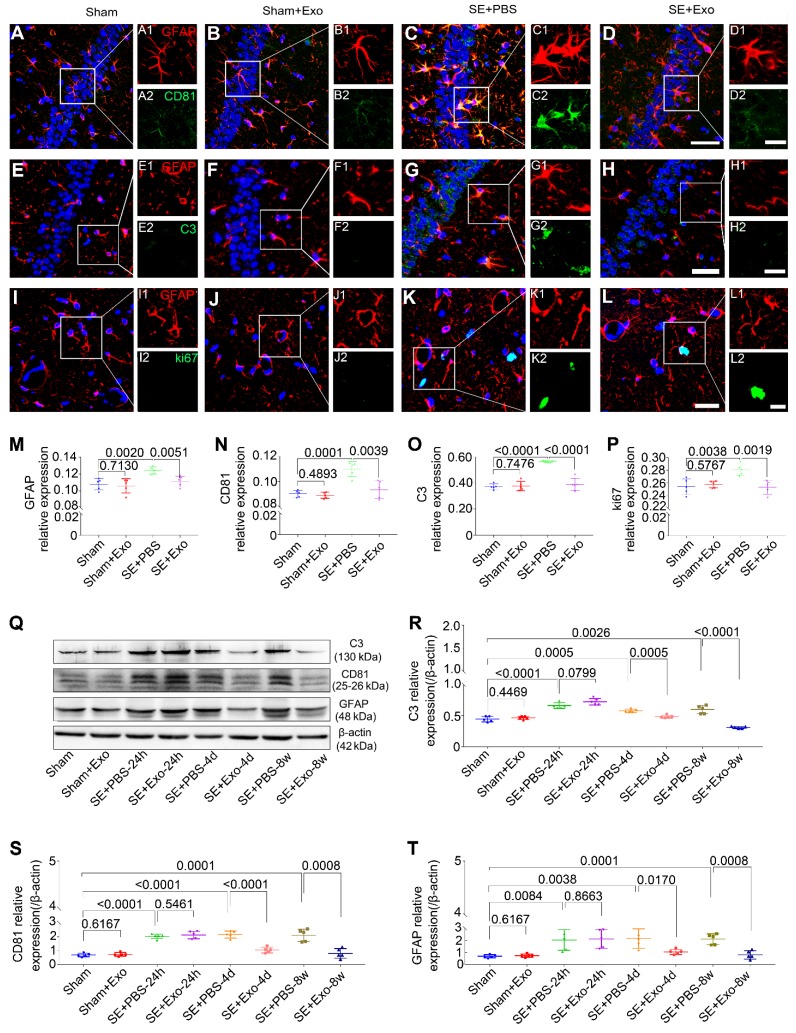
** Intraventricular injection of MSC-Exo alleviates SE-induced hippocampal astrocytic activation in mice.** The influence of MSC-Exo on hippocampal astrocytic activation was examined in the acute and chronic stages of SE. (A-L) Representative images display the immunofluorescence intensity of GFAP (A-L, red), CD81 (A-D, green), C3 (E-H, green), and ki67 (I-L, green) in the hippocampus at 4 d post-SE, cell nuclei stained with DAPI (blue). (M-P) Fluorescence intensity assay of the relative expression of GFAP (M), CD81 (N), C3 (O), and ki67 (P) in each group. (Q) Western blots of protein expression of C3, CD81, and GFAP at 24 h, 4 d, 7 d, and 8 w post-SE in the hippocampus. (R-T) Statistical analysis of changes in C3 (R), CD81 (S), and GFAP (T) expression in each group. Statistical difference between groups is displayed in histograms. Scale bars: A-L, 50 μm; A1-L1 and A2-L2, 20 μm.

We used Western blotting to examine the anti-reactive astrogliosis of MSC-Exo using pilocarpine-induced SE model, which is characteristic of robust reactive astrogliosis in the hippocampus. Hippocampal tissues were collected from the Sham, Sham+Exo, SE+PBS, and SE+Exo experimental groups (Figure 5Q). Mice receiving PBS only had a significant increase in C3 (Figure 5R, *P* < 0.01), CD81 (Figure 5S, *P* < 0.01) and GFAP (Figure 5T, *P* < 0.01) expression 24 h, 4 d, and 8 w post-SE. However, the MSC-Exo treatment reduced the marker expression 4 d and 8 w post-SE (Figure 5R-T, *P* < 0.05), but not 24 h post-SE (Figure 5R-T, *P* > 0.05). The putative therapeutic actions of MSC-Exo on reactive astrogliosis were further examined using immunohistochemistry 4 d post-SE (Figure 5A-L). Fluorescence intensity analysis (object area of immuno-reactive cells in % / pre-determined area of interest) showed a remarkable decrease in the relative expression of GFAP, CD81, C3, and ki67 (Figure 5M-P) in the SE+Exo group compared to the SE+PBS group (*P* < 0.01). Importantly, no adverse effects of MSC-Exo on animals in the Sham group were seen (Figure 5M-P and R-T, *P* > 0.05). Thus, the data showed that intraventricular injections of MSC-Exo reduce the hippocampal reactive astrogliosis in a mouse model of SE.

### MSC-Exo attenuates SE-induced hippocampal inflammatory response in mice

**Figure 6:**
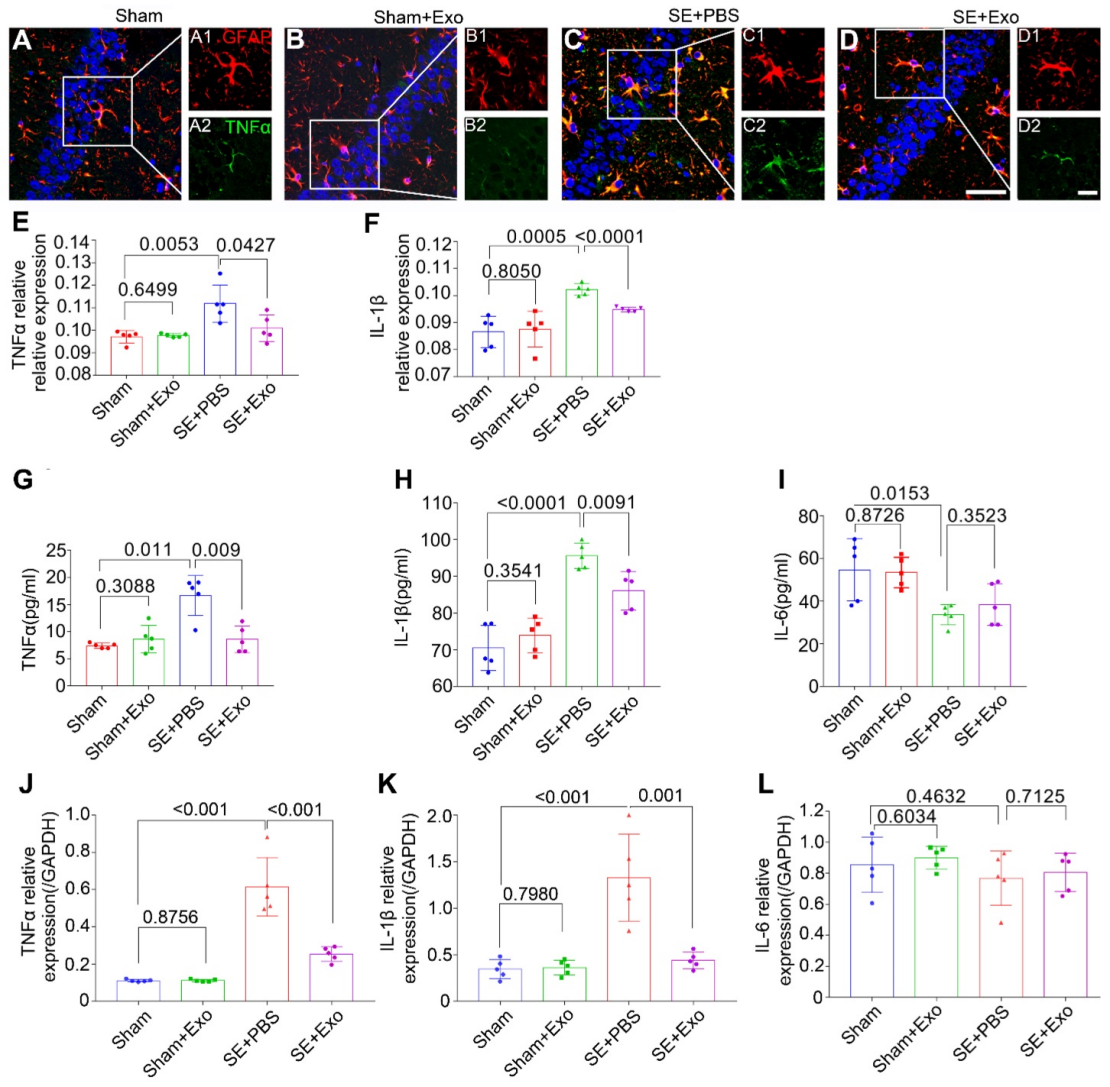
** MSC-Exo injection attenuates SE-induced hippocampal inflammatory responses.** After the SE model received for 4 d, the inflammatory responses were investigated in SE models 4 d after MSC-Exo administration. (A-D) Hippocampal tissues double-stained with GFAP and TNFα in each group, magnified images on the right are of GFAP+ (red, A1-D1) and TNFα+ (green, A2-D2) cells, cell nuclei stained with DAPI (blue). (E-F) Fluorescence intensity of the relative expression of TNFα (E) and IL-1β (F) in the whole hippocampus including CA1, CA2, CA3, and DG subareas of each group. (G-I) Histograms of the concentration of inflammatory cytokines including TNFα (G), IL-1β (H), and IL-6 (I) in each group by ELISA. (J-L) qPCR of the RNA levels (relative expression to GAPDH) of *TNFα* (J), *IL-1β* (K), and *IL-6* (L) in the hippocampal tissues of the experimental groups. Statistical difference between groups is shown in all histograms. Scale bars: A-D, 50μm; A1-D1, 20μm; A2-D2, 20μm.

To examine the anti-inflammatory effects of MSC-Exo in the SE model, bilateral hippocampal tissues were collected 4 d post-SE and processed for immunohistochemistry, ELISA, and qPCR. Results are displayed in Figure 6A-L. Since inflammatory cytokines such as TNFα, IL-1β, and IL-6 can be secreted by microglia in addition to the astrocytes, double immunostaining was carried out to determine their expression patterns in the hippocampus. Results showed that GFAP was co-expressed with TNFα (Figure 6A-D) and IL-1β (not shown), and the fluorescence intensity of TNFα and IL-1β markedly increased after the pilocarpine injection (Figure 6E-F, *P* = 0.01), whereas MSC-Exo treatment reduced their expression levels compared to the SE+PBS group (Figure 6E-F, *P* < 0.05). Similarly, the concentration of proinflammatory cytokines TNFα (Figure 6G) and IL-1β (Figure 6H) in the hippocampus showed a remarkable increase (*P* < 0.05) by ELISA, but the IL-6 showed an increase compared to the Sham group (Figure 5I, *P* = 0.0153). MSC-Exo treatment significantly decreased the production of proinflammatory cytokines TNFα (Figure 6G, *P* = 0.009) and IL-1β (Figure 6H, *P* < 0.0091) compared to the SE+PBS group. Furthermore, *TNFα*, *IL-1β,* and *IL-6* (Figure 6J-L) gene expression in the hippocampus was detected by qPCR. Our data showed that pilocarpine treatment increased *TNFα* and *IL-1β* gene expression in the hippocampus compared to the Sham group (Figure 6J, K, *P* < 0.001), whereas MSC-Exo administration significantly decreased *TNFα* and *IL-1β* RNA levels compared to the SE+PBS group (Figure 6J, K, *P* < 0.01). There was no statistical difference between Sham and Sham+Exo group in the qPCR measured *IL-6* expression, which was consistent with ELISA results (Figure 6I, L). These data suggested that MSC-Exo can attenuate the SE-induced hippocampal inflammatory responses in mice.

### MSC-Exo treatment restores SE-induced hippocampal astrocyte activation via Nrf2-NF-κB signaling pathway in mice

**Figure 7:**
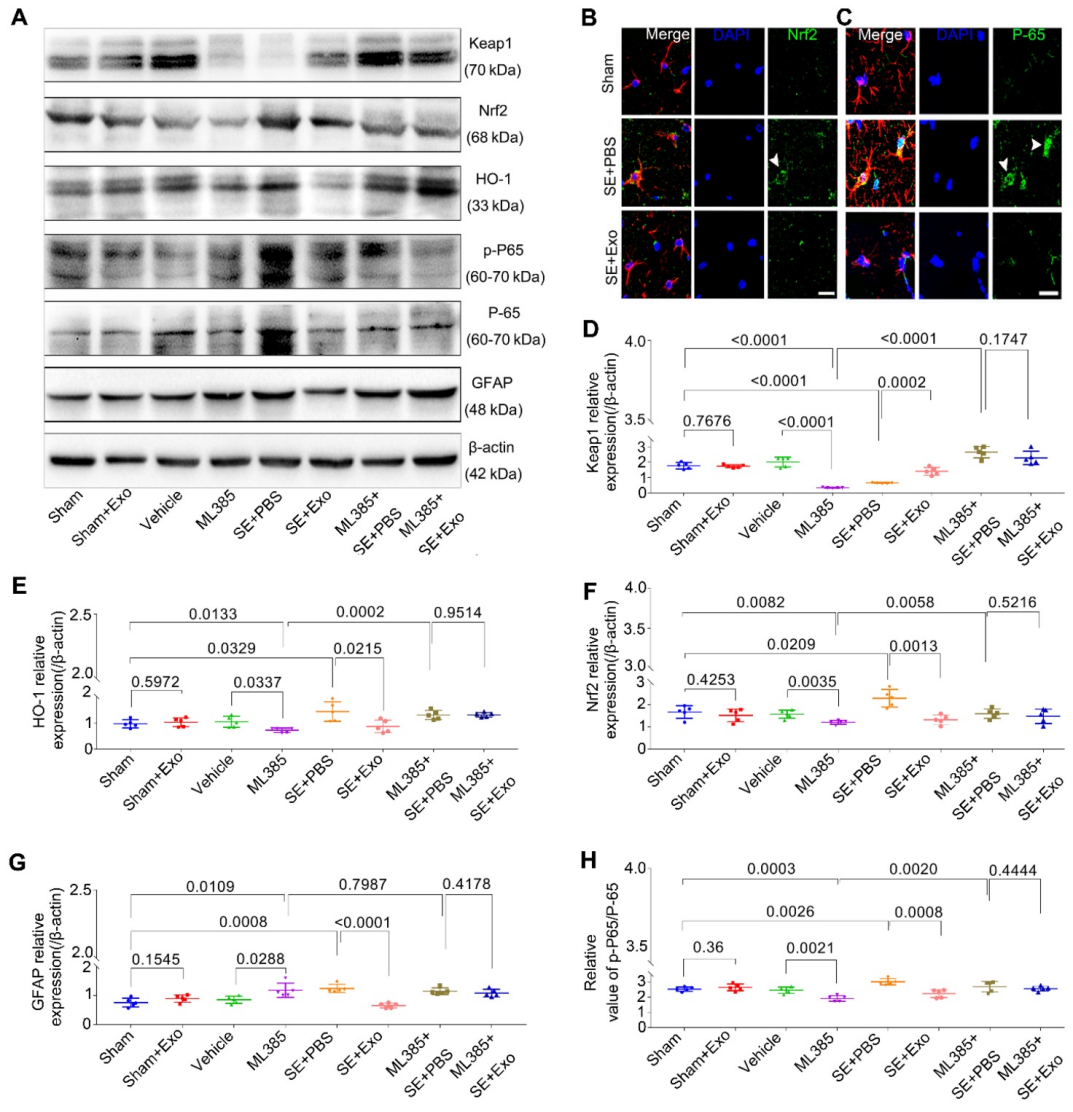
** ML385 (Nrf2 inhibitor) injection reverses inhibition of MSC-Exo on hippocampal astrocytic activation *in vivo*.** ML385 (Nrf2 inhibitor) injection was used to explore the mechanism of MSC-Exo in anti-inflammation *in vivo*. (A) Western blots of antioxidant and anti-inflammatory markers in hippocampal tissues before and after ML385 injection. (B-C) Representative images of the nuclear translocation of Nrf2 (B, arrowhead) and P-65 (C, arrowheads) in Sham, SE+PBS, and SE+MSC-Exo groups. (D-H) Statistical analysis of the relative expression of Keap1 (D), HO-1 (E), Nrf2 (F), GFAP (G), and p-P-65 / P-65 (H) in the hippocampus. Statistical difference between groups is shown in all histograms. Scale bars: B and C, 30μm

To investigate whether the Nrf2-NF-κB signaling pathway was involved in the inhibition of astrocyte activation, we examined hippocampal tissues 4 d post-SE using Western blotting and immunohistochemistry. Pilocarpine treatment significantly increased GFAP, Nrf2, p-P65/P-65, and HO-1 expression in the hippocampus but reduced Keap1 expression (Figure 7D-E,* P* < 0.05). Also, it promoted the nuclear translocation of the Nrf2 (Figure 7B) and P-65 (Figure 7C) in the SE mice. A comparison of MSC-Exo treatment (SE+Exo group) vs. PBS (SE+PBS group) revealed that MSC-Exo could reverse the oxidation (Figure 7B, E and F) and inflammation (Figure 7C, G and H) phenotypes. No significant differences were observed between Sham+Exo and Sham group (Figure 7D-H, *P* > 0.05) indicating that MSC-Exo treatment alone did not alter the expression of markers associated with these phenotypes.

To explore the mechanism of Nrf2-NF-κB signaling pathway involved in the inhibition of astrocyte activation *in vivo*, we used ML385, an inhibitor of Nrf2. Protein assays showed that mice treated with ML385 had a significant decrease in Keap1, HO-1, Nrf2, and p-P65/P-65 (Figure 7D-H, *P* < 0.05) expression compared to Sham or Vehicle control groups. Although these markers were upregulated following the SE induction (Figure 7D-F and H, *P* < 0.01), no significant differences were observed between ML385+SE+PBS and ML385+SE+Exo groups (Figure 7D-F and H, *P* > 0.05). Furthermore, Nrf2 inhibition resulted in increased GFAP expression compared to Sham or Vehicle groups (Figure 7G, *P* < 0.05) which was not present in the ML385+SE+Exo group treated with MSC-Exo (Figure 7G, *P* > 0.05). These findings suggest that MSC-Exo treatment can restore the LPS- and SE-induced hippocampal astrocyte activation in mice via the Nrf2-NF-κB signaling pathway.

### MSC-Exo treatment restores SE-induced learning and memory impairment

**Figure 8:**
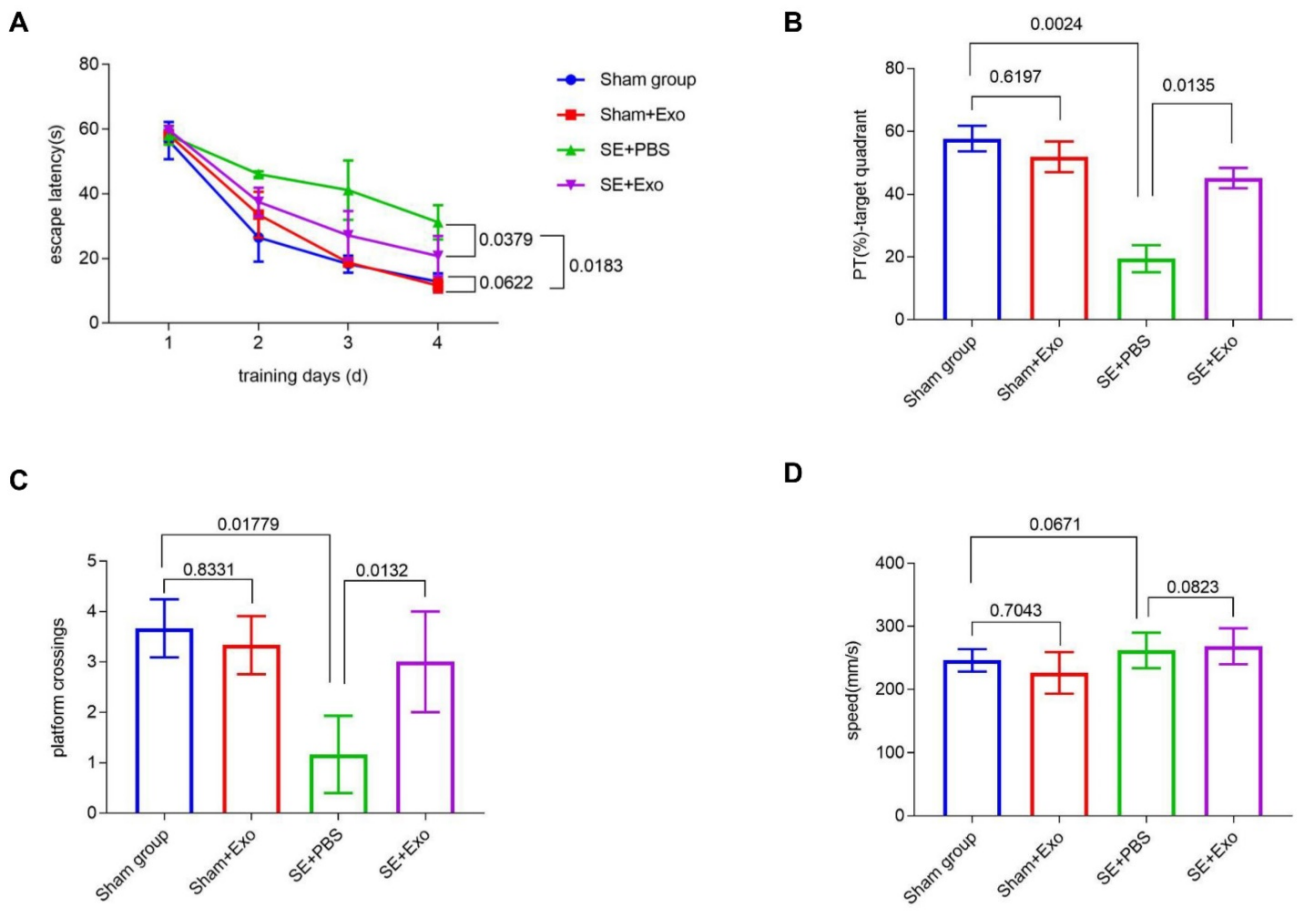
** MSC-Exo treatment improves SE-induced learning and memory impairments in mice.** Learning and memory impairments of each group were tested using Morris water maze. (A) Escape latency of each group within the training days. (C-D) Histograms of the PT (%)-target quadrant (B), platform crossings (C), and speed (mm/s) (D) in different experimental groups. Statistical difference between groups is shown in the graph and histograms.

We investigated the behavioral effects of SE induction using the Morris Water Maze learning and memory paradigm consisting of four training sessions. Repeated measures ANOVA showed no difference in the first learning session between each group. During the hidden platform tasks, SE mice showed a significant (*P* = 0.00183) decrease in the escape latency compared to the Sham group (Figure 8A). Administration of MSC-Exo reversed this tendency (Figure 8A, *P* = 0.0379). After 24 h, memory encoding and retrieval in each group was tested by recording the percentage of time spent in the target quadrant and counting platform crossings. Compared to the Sham group, SE mice showed a marked decrease in % of total swim distance in target quadrant (PT) (*P* = 0.0024) and platform crossings (*P* = 0.01779) (Fig 8B and C). Reduction of the learning and memory impairment in the SE+Exo group was shown by the increased percentage of time spent in the target quadrant (Figure 8B, *P* = 0.0135) and the platform-crossings (Figure 8C, *P* = 0.0132) in comparison with the SE+PBS group. There was no difference in swim speed between the groups.

## Discussion

Our study showed that in mice with pilocarpine-induced SE, MSC-Exo treatment ameliorated inflammation-induced astrocytic alterations including reactive astrogliosis, inflammatory responses, aberrant calcium signaling, and mitochondrial dysfunction, and reduced learning and memory impairment. Furthermore, our data indicated that MSC-Exo could restore A1 astrocyte activation via regulation of Nrf2-NF-κB signaling pathway, suggesting that MSC-Exo can be a potential nanotherapeutic agent for the treatment of neurological diseases whose pathological basis entails hippocampal astrocytic alterations.

Neuroinflammation is emerging as a central pathological process in many neuropsychiatric disorders either as a causative factor or as a secondary response to a CNS insult. Activated microglia can release both pro-inflammatory (M1) and anti-inflammatory (M2) factors that have been shown to be both beneficial and deleterious in neuroinflammatory processes 38. Astrocytes exhibit a similar phenotype to microglia (A1 and A2) 14 and immune responses to proinflammatory stimuli and injury. However, it has been shown that astrogliosis is often more persistent than microgliosis and is believed to be more significant in amplifying inflammatory processes and thereby inducing greater damage 39-41. Astrocytes have been shown to play a crucial role in regulating and restricting CNS inflammation 42, 43. Thus, protecting astrocytes from inflammatory stimuli or ameliorating inflammation-induced astrocyte alterations could be a promising therapeutic strategy for many neurological diseases. Although immunomodulatory properties of MSCs have shown promise in the treatment of inflammatory disorders in various organs including brain, current evidence suggests that MSC-Exo have a greater therapeutic potential than MSCs; they carry many anti-inflammatory agents such as mRNAs, microRNAs and proteins, are less immunogenic, and are easier to store and administer for therapeutic purposes than MSCs 7, 44. In this study, we found that MSC-Exo could be incorporated into hippocampal astrocytes *in vitro* and *in vivo*, an observation that has a considerable significance for the future development of MSC-Exo-based therapeutic agents targeting hippocampal astrocytes.

Reactive astrogliosis is a pathologic hallmark of neurological injury 12. Therefore, targeting reactive astrocytes could be an effective therapeutic strategy for epilepsy and other neurodegenerative diseases 12, 20. Generally, compared to oligodendrocytes and neurons, astrocytes maintain high intracellular concentrations of antioxidants making these cells resistant to inflammation and oxidative stress 45. However, persistent reactive astrogliosis stimulates proinflammatory cytokine production and release and contributes to the spread of gliosis and neuronal loss following inflammatory stimulation 12, 45. Neuroinflammation and ischemia can induce two different types of reactive astrocytes known as A1 and A2. The A1 astrocytes lose most of the normal astrocyte functions and have been shown to be toxic to neurons or destructive to synapses 14. Therefore, attenuation of reactive astrogliosis or transformation of A1 astrocytes back to their pre-astrogliosis state may be an important strategy for preventing the inflammatory injury associated with CNS diseases.

In this study we investigated, both *in vitro* and *in vivo,* whether MSC-Exo administration decreases the expression of GFAP (astroglial marker), C3 (A1 astrocyte marker), and ki67 (cell proliferation marker) in the LPS-stimulated cultured primary hippocampal astrocytes and in SE-induced hippocampus. Analysis of immunofluorescence intensity and alterations in protein levels revealed that MSC-Exo could prevent astrocytes from inflammation-induced reactive astrogliosis manifested by an abnormal increase in the number of astrocytes and hypertrophy of soma. Reduced expression of CD81, a regulator of astrocytic activation 15, 16 was also detected after administration of MSC-Exo in* vitro* and *in vivo.* To our knowledge, this is the first demonstration of MSC-Exo regulation of the SE-induced reactive astrocytes associated with the tetraspanin superfamily member CD81 expression in mice. Furthermore, since the hippocampal tissue is highly susceptible to SE, water maze learning and memory tests can serve as a reliable tool for identifying the behavioral effects of a hippocampal injury 46. Here, we showed that impaired spatial learning and memory retrieval post-SE were restored by MSC-Exo treatment. These results suggest that MSC-Exo treatment can ameliorate the inflammation-induced alterations in astrocytes as well as the resultant behavioral dysfunction.

LPS acts as the prototypical endotoxin in many cell types including macrophages, monocytes, and dendritic cells in which it promotes the secretion of pro-inflammatory cytokines (e.g., TNFα, IL-1β, and IL-6), nitric oxide, and eicosanoids. LPS is a classical stimulus that induces an inflammatory response in astrocytes and is used to investigate underlying molecular or cellular mechanisms of neurological diseases 23, 47. Here, we used LPS to stimulate the primary culture of hippocampal astrocytes and found a dramatic increase in TNFα and IL-1β expression that was consistent with a previous report 48. As expected, MSC-Exo significantly reduced the upregulation of these proinflammatory cytokines. These results indicated that MSC-Exo attenuated the inflammatory responses of the primary cultured hippocampal astrocytes stimulated by LPS. A significant role of inflammatory and immune mediators has been well documented in the initiation of seizures as well as epileptogenesis 45. Importantly, a proinflammatory response induced by temporal lobe epilepsy in the brain is mainly characterized by glial activation 45, 49. Our experiments showed that the post-SE MSC-Exo treatment significantly reduced TNFα and IL-1β protein levels and gene expression in hippocampus together with the astroglial marker GFAP that is co-expressed with these proinflammatory cytokines. We did not detect a significant difference, both *in vitro* and *in vivo*, in IL-6, a cytokine that has both proinflammatory and anti-inflammatory properties 50; however, our results indicated that administration of MSC-Exo could reduce the inflammatory responses associated with the hippocampal astrocyte activation.

Recently, it has been reported that MSC-Exo exert therapeutic effects by reducing astrocytic activation in spinal cord injury 51, 52, but the restoration by MSC-Exo on inflammation-induced aberrant calcium signaling and mitochondrial dysfunction in astrocytes has not been reported previously. Neuronal calcium signaling within the cytosol and endoplasmic reticulum has been well documented 53, but other CNS-resident cell types such as astrocytes affected by aberrant calcium signaling have not been thoroughly studied 11. It has been proposed that gliotransmitters, such as ATP, are released through a Ca^2+^-dependent mechanism that can modulate neuronal excitability and synaptic transmission 54, 55. Our calcium imaging results showed an increase in Ca^2+^ influx after ATP stimulation of LPS-induced primary cultured hippocampal astrocytes, suggesting that LPS induction can alter the calcium signaling of the hippocampal astrocytes. Altered calcium signaling drives many pathophysiological processes associated with aging, neurodegenerative diseases, or epilepsy 56, 57. Inflammatory stimuli, such as LPS, regulate molecular pathways in astrocytes that are associated with immune- and injury-related functions and significantly alter calcium signaling stimulated by multiple G-protein-coupled and ionotropic (e.g. N-methyl-D-aspartate, NMDA) receptors 58. These receptors mediate calcium fluxes large enough to trigger a substantial initial increase in cytosolic free calcium concentration in astrocytes 48, 58, while calcium channel blockers can inhibit LPS-induced astrocyte activation and inflammatory response, suggesting that these calcium fluxes could indicate LPS-induced astrocyte alterations 23, 53.

In this study, we demonstrated that MSC-Exo reversed altered calcium signaling in LPS-stimulated primary hippocampal astrocytes by decreasing the Ca^2+^ influx. These results can be interpreted to suggest that MSC-Exo restore the calcium concentration in LPS-induced primary cultured astrocytes, but detailed mechanisms of this restoration require further studies. Besides their energy-generating function, mitochondria participate in calcium homeostasis and cellular signaling by generating reactive oxygen species (ROS), which plays a role in cellular survival 45. Mitochondria act as local Ca^2+^ buffers to the adjacent Ca^2+^ release sites such as the endoplasmic reticulum or plasma membrane Ca^2+^ channels 55. We used JC-1 as a mitochondrion-specific lipophilic cationic fluorescence dye to probe the effect of MSC-Exo on mitochondrial dysfunction in LPS-induced primary hippocampal astrocytes. Changes in the MMP lead to alterations of mitochondrial function and play a role in the regulation of apoptotic cell death in the intrinsic pathway 24. In our study, JC-1 staining showed that a significant reduction of JC-1 ratio (as well as restoration of mitochondrial membrane potential) in LPS-stimulated primary hippocampal astrocytes could be improved by MSC-Exo treatment suggesting its therapeutic effect on mitochondrial dysfunction as well as LPS-induced aberrant calcium signaling in hippocampal astrocytes. To the best of our knowledge, these results provide the first evidence that MSC-Exo ameliorate LPS-induced aberrant calcium signaling and mitochondrial dysfunction in cultured primary hippocampal astrocytes.

Inflammation and oxidative stress are the two sides of the same coin in neurological diseases such as epilepsy and Parkinson's disease 40, 59. Nrf2 is a known regulator of oxidative damage triggered by injury and inflammation 60, 61, while NF-κB is a protein complex that controls cytokine production and cell survival 62. Therefore, an interplay between Nrf2 and NF-κB is believed to be responsible for regulating processes leading to neuroinflammation and oxidative stress 19. Herein, we showed that increased expression of Nrf2, HO-1 and p-P65/P-65 and nuclear translocation of Nrf2 and NF-κB could be induced by LPS stimulation *in vitro*. Similarly, pilocarpine injection also resulted in Nrf2-NF-κB signaling activation *in vivo*. Nrf2 is not degraded directly under inflammation or oxidative stress, but instead, it translocates to the nucleus where it binds to a DNA promoter and initiates transcription of antioxidative genes and synthesis of their corresponding proteins 63. NF-κB activation drives immunological responses including glial proliferation, leukocyte infiltration, and proinflammatory cytokine production, which can be reflected in the altered ratio of p-P65/P-65 as the subunits of NF-κB P-65 translocate to the cell nuclei 18, 25, 64. Besides, Nrf2 is retained in the cytoplasm by Keap1 that degrades Nrf2 by ubiquitination, and HO-1 is an Nrf2 target gene that has been shown to protect cells 65. A recent study showed that Nrf2 alone could be an independent regulator of oxidation or serve as the master regulator of the antioxidant response element 66; decreased expression of Keap1 in SE mice may, therefore, be attributed to the off-target effects or competitive inhibition by Nrf2. These results indicate that the Nrf2-NF-κB signaling pathway is activated in response to inflammatory stimulation.

Our in vivo and in vitro data showed that MSC-Exo could reverse hippocampal astrocyte oxidation (e.g. upregulation and nuclear translocation of Nrf2) and inflammation (*e.g.* NF-κB activation and translocation, increased GFAP expression) phenotypes. Together these results suggested that the Nrf2-NF-κB signaling pathway was involved in the inhibition of astrocytic activation by MSC-Exo. Furthermore, both the sgRNA knockdown of *Nrf2* and Nrf2 inhibition showed downregulation of Keap1, HO-1 and p-P65/P-65 and upregulation of GFAP *in vitr*o and *in vivo* suggesting their regulation by Nrf2 in hippocampal astrocytes. Notably, even though the results indicated that LPS stimulation or pilocarpine could activate Nrf2-NF-κB signaling in the genetically engineered cells or animal models, the expression of Nrf2, Keap1, HO-1, p-P65/P-65 and GFAP could not be reversed by MSC-Exo administration *in vitr*o and *in vivo*. Together, these results indicated that MSC-Exo could attenuate the inflammation-induced astrocytic activation by regulating the Nrf2-NF-κB signaling pathway.

In conclusion, our study shows that MSC-Exo is a promising nanotherapeutic agent for amelioration of inflammation-induced astrocytic alterations and Nrf2-NF-κB signaling pathway plays an important role in regulating astrocyte activation in mice.

## Supplementary Material

Supplementary figures.

## Abbreviations

AD: Alzheimer's disease
α-MEM: α-minimum essential medium
ACSF: artificial cerebrospinal fluid
ATP: adenosine triphosphate
BCA: Bicinchoninine acid
CCK-8: Cell Counting Kit-8
CCM: complete culture medium
CM-A954: C5 Maleimide-Alexa 594
CNS: central neural system
DAPI: 4', 6-diamidino-2-phenylindole
DG: dentate gyrus
DMEM: Dulbecco's Modified Eagle Medium
ELISA: enzyme-linked immunosorbent assays
EVs: extracellular vesicles
FBS: fetal bovine serum
GAPDH: gyceraldehyde phosphate
GFAP: glial fibrillary acidic protein
GPC: G-protein-coupled
HO-1: hemeoxygenase 1
IL-1β: interleukin-1 receptor
IL-6: interleukin- 6
Keap1: kelch-like ECH-associated protein 1
LPS: lipopolysaccharides
MMP: mitochondrial membrane potential
MSC-Exo: mesenchymal stem cell-derived exosomes
NMDA: N-methyl-D-aspartic acid
Nrf2: nuclear factor (erythroid-derived 2)-like 2
OD: optical density
PBS: phosphate buffer saline
P65: nuclear factor-kappaB 65
p-P65: phosphor NF-κBp-65
qPCR: real-time Quantitative PCR
ROS: reactive oxygen species
SE: status epilepticus
TNFα: tumor necrosis factor*-α*
TBST: Tris Buffered saline Tween
