# Supplementary material for: Mesenchymal stem cell-derived exosomes as a nanotherapeutic agent for amelioration of inflammation-induced astrocyte alterations in mice
Source: Theranostics. 2019 Aug 14;9(20):5956–75. doi: 10.7150/thno.33872 (PMC6735367; doi:10.7150/thno.33872)
Supplement: Supplementary file 1 — Supplementary figures. [file thnov09p5956s1.pdf]

Figure S1. Cell viability of primary cultured hippocampal astrocytes after LPS or MSC-Exo administration using CCK-8 assay

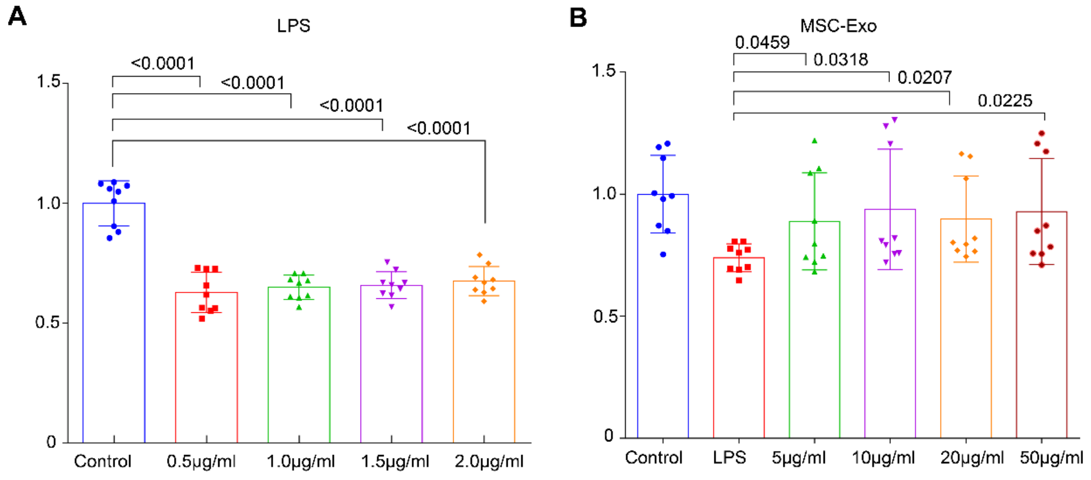

(A) Histograms of different concentrations of LPS-induced cytotoxicity on the primary culture of hippocampal astrocytes. (B) Dose-response effects of MSC-Exo on cell viability in LPS-induced primary cultured astrocytes.

Figure S2. Dose-response effects of MSC-Exo on inflammation-induced astrocytic activation *in vitro* and *in vivo*

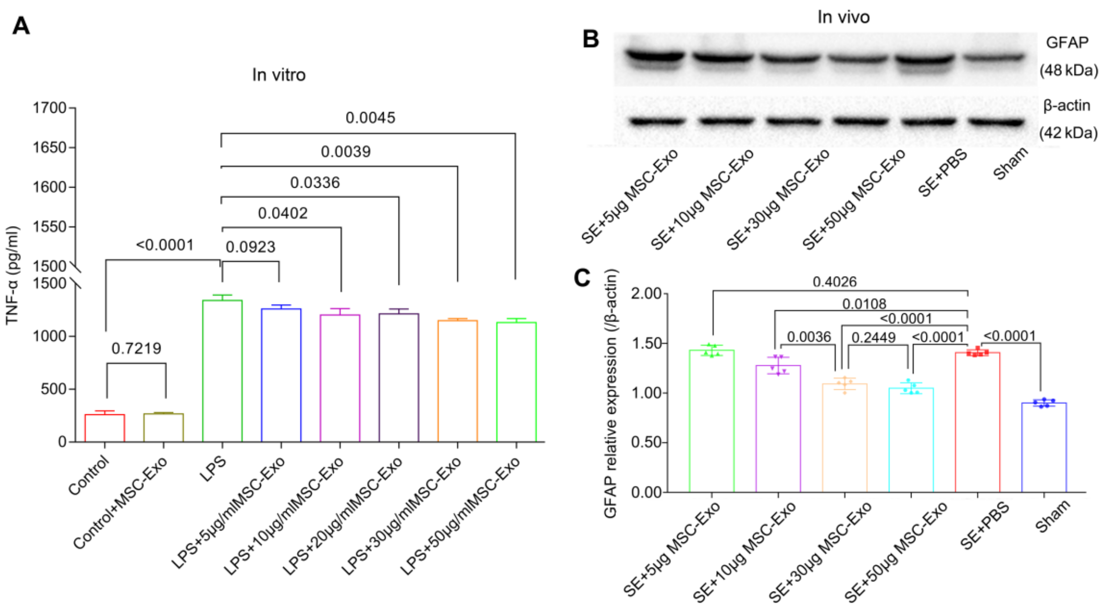

MSC-Exo effects on TNFα and GFAP relative expression in LPS-stimulated astrocytes *in vitro* (A) and SE mice *in vivo* (B and C). Note a linear decrease in TNFα expression with increasing doses of MSC-Exo (A) and a similar linear decrease of GFAP expression in SE mice (B, C). Panel B shows Western blotting results corresponding to each column in C. 10 μg/ml (*in vitro*) and 30 μg/per mouse (*in vivo*) MSC-Exo was employed in this study.
